# Interferon Alpha Favors Macrophage Infection by Visceral *Leishmania* Species Through Upregulation of Sialoadhesin Expression

**DOI:** 10.3389/fimmu.2020.01113

**Published:** 2020-06-09

**Authors:** Lieselotte Van Bockstal, Dimitri Bulté, Magali Van den Kerkhof, Laura Dirkx, Dorien Mabille, Sarah Hendrickx, Peter Delputte, Louis Maes, Guy Caljon

**Affiliations:** Laboratory of Microbiology, Parasitology and Hygiene (LMPH), Faculty of Pharmaceutical, Biomedical and Veterinary Sciences, University of Antwerp, Wilrijk, Belgium

**Keywords:** *Leishmania*, sialoadhesin, CD169, macrophages, IFN-α, type I IFN

## Abstract

Type I interferons (IFNs) induced by an endogenous *Leishmania* RNA virus or exogenous viral infections have been shown to exacerbate infections with New World Cutaneous *Leishmania* parasites, however, the impact of type I IFNs in visceral *Leishmania* infections and implicated mechanisms remain to be unraveled. This study assessed the impact of type I IFN on macrophage infection with *L. infantum* and *L. donovani* and the implication of sialoadhesin (Siglec-1/CD169, Sn) as an IFN-inducible surface receptor. Stimulation of bone marrow-derived macrophages with type I IFN (IFN-α) significantly enhanced susceptibility to infection of reference laboratory strains and a set of recent clinical isolates. IFN-α particularly enhanced promastigote uptake. Enhanced macrophage susceptibility was linked to upregulated Sn surface expression as a major contributing factor to the infection exacerbating effect of IFN-α. Stimulation experiments in Sn-deficient macrophages, macrophage pretreatment with a monoclonal anti-Sn antibody or a novel bivalent anti-Sn nanobody and blocking of parasites with soluble Sn restored normal susceptibility levels. Infection of Sn-deficient mice with bioluminescent *L. infantum* promastigotes revealed a moderate, strain-dependent role for Sn during visceral infection under the used experimental conditions. These data indicate that IFN-responsive Sn expression can enhance the susceptibility of macrophages to infection with visceral *Leishmania* promastigotes and that targeting of Sn may have some protective effects in early infection.

## Introduction

Leishmaniasis is a family of related protozoal diseases occurring in the New- and Old World and is caused by *Leishmania* parasites responsible for clinical features ranging from cutaneous, mucocutaneous to visceral manifestations. Visceral leishmaniasis (VL), also known as kala-azar, is a lethal neglected tropical disease caused by *Leishmania donovani* and *L. infantum* and responsible for ~0.2–0.4 million cases each year (1). It is a vector-borne disease transmitted by the bites of infected female phlebotomine sand flies (2). In the vertebrate host, entry and survival inside myeloid cells are essential factors to complete its life cycle (3) and to enable dissemination to internal organs such as the liver, spleen and bone marrow (4).

Recent reports on New World cutaneous *L. guyanensis* infections revealed a considerable impact of exogenous IFN-inducing viruses and an endogenous *Leishmania* dsRNA virus (LRV1) on primary infection and reactivation in mice (5). LRV1 presence in clinical isolates of *L. braziliensis* has been associated to increased risk of treatment failure (6). LRV-sequences were also detected in an Iranian *L. infantum* clinical isolate from a patient unresponsive to antimonial treatment (7). Viral co-infections and presence of *Leishmania* RNA virus are therefore increasingly perceived as risk factors for pathogenicity of human leishmaniasis (5, 7–9). The virus appears to use the *Leishmania* exosomal pathway to reach the extracellular environment (10). The exacerbating features of LRV1 in *L. guyanensis* were linked to the induction of type I interferons which primarily occurred through stimulation of the endosomal Toll-Like Receptor 3 (TLR3) pathway by viral dsRNA in mice (11, 12). Type I IFN is known to trigger the expression of various so-called interferon-stimulated genes (ISGs) (13), including some that are involved in viral recognition and entry. Sialoadhesin (Sn, CD169, Siglec-1) is an ISG-gene product (14–17) expressed on macrophages, belonging to the Siglec (sialic acid binding Ig-lectin) family (18). Human and mice Sn share 72% sequence homology (19, 20) and, unlike other Siglecs, seem to lack tyrosine-based signaling motifs which suggests a primary role in cell-cell interactions rather than in cell signaling (21). During HIV-infection, Sn expression levels have been correlated with type I IFN levels and inflammatory disease progression *in vivo* in macaques (16). Regarding HIV-1, Pino et al. showed that IFN-α activated macrophages have an enhanced ability to capture HIV-1 via Sn recognition. These macrophages could fuel novel CD4^+^ T cell infections and contribute to HIV-1 dissemination (22). Recent reports described that Sn recognizes the sialic acid moieties onto the *Leishmania* surface (3, 23, 24) and is responsible for phagocytosis during a *Leishmania* infection (3). Other pathogens such as *Campylobacter jejuni* (25), group B *Streptococcus* (26) and *Trypanosoma cruzi* (27) were also shown to be phagocytosed by macrophages using the Sn-sialic acid interaction. Sn becomes highly upregulated under conditions of IFN-α stimulation, for example, during viral or bacterial infections *in vitro* and *in vivo* (14, 16, 17). As such, co-infections and/or IFN-α stimulation may have an impact on the course and pathogenicity of a *Leishmania* infection (5).

The present study evaluated the role of type I IFN in the infection outcome of different *L. infantum* and *L. donovani* strains. IFN-α stimulated macrophages showed higher infection levels compared to control-treated macrophages. Since Sn expression was described to be upregulated by type I IFN, we further unraveled the role of Sn during *in vitro* and *in vivo Leishmania* infections.

## Materials and Methods

### Ethics

The use of laboratory rodents was carried out in accordance to all mandatory guidelines (EU directives, including the Revised Directive 2010/63/EU on the Protection of Animals used for Scientific Purposes that came into force on 01/01/2013, and the declaration of Helsinki in its latest version) and was approved by the ethical committee of the University of Antwerp, Belgium (UA-ECD 2014-17, UA-ECD 2017-04, UA-ECD 2015-90).

### Animals

Female C57BL/6 and BALB/c mice (6–8 weeks old) were used for the collection of bone marrow cells and for the *in vivo* bioluminescent imaging experiments. Female golden hamsters (body weight 100-120 g) were used as donors for the collection of *Leishmania* amastigotes. Animals were purchased from Janvier (France) and kept in quarantine for at least 5 days before starting the experiment. Sn-deficient mice (Sn^−/−^ C57BL/6) were reared at our facilities from breeding pairs provided by UGent (Prof. Dr. Dirk Elewaut, Molecular Immunology and Inflammation Unit, VIB-UGent). Food for laboratory rodents and drinking water were available *adlibitum*.

### Parasite Species/Strains

The different *L. infantum, L. donovani*, and *L. major* strains used in this paper for the *in vitro* infections are listed in Table 1. Promastigotes were routinely cultured in T25 culture flasks containing 5 mL of HOMEM medium (Invitrogen, UK) supplemented with 10% heat inactivated fetal bovine serum (iFBS). *Ex vivo* amastigotes of *L. infantum* ITMAP263 were obtained from the spleen of heavily infected donor hamsters and purified using two centrifugation steps as described elsewhere (28). The strains for the *in vivo* infections were the bioluminescent *L. infantum* MHOM/FR/96/LEM3323^PpyRE9^ and MHOM/MA/67/ITMAP263^PpyRE9^, generated by the stable introduction of the red-shifted firefly luciferase PpyRE9 using the pLEXSY-hyg2.1 vector (29).

**Table 1:** Overview of *Leishmania* isolates used and their respective origin.

**Strain**	**Code**	**Origin**
***L. infantum***		
ITMAP263	MHOM/MA/67/ITMAP263	Reference lab strain, originally isolated from VL-patient in Morocco
LEM3323	MHOM/FR/96/LEM3323	French field isolate from HIV-patient
LEM5159	MHOM/FR/2006/LEM5159	French field isolate from HIV-patient
LLM2346	MHOM/ES/2016/LLM-2346	Spanish field isolate
***L. donovani***		
Ldl82	MHOM/ET/67/L82	Reference lab strain, originally isolated from VL-patient in Ethiopia
LLM1599	MHOM/ET/2007/LLM-1599	Ethiopian field isolate
LLM1600	MHOM/ET/2007/LLM-1600	Ethiopian field isolate
***L. major***		
JISH118	MHOM/SA/85/JISH118	Saudi Arabian field isolate

### Purification of a Sn-Specific Bivalent Nanobody and Conventional Antibody

Anti-mouse Sn monoclonal antibodies (mAb) SySy94 were produced and purified as described previously (17). Anti-mouse Sn bivalent nanobody (Biv4.40 Nb, courtesy UGent) was produced in the periplasm of transformed *Escherichia coli*. The bacterial expression clone was expanded in LB Broth medium containing 100 μg/mL ampicillin, 2 mM MgCl_2_ and 0.1% D-glucose at 37°C and 200 rpm in a New Brunswick incubator shaker. The induction of protein synthesis was carried out at OD_600_ 0.6–0.9 by addition of 1 mM IPTG (isopropyl β- d-1-thiogalactopyranoside) and continued incubation at 28°C and 200 rpm. Bacterial pellets were collected by centrifugation (20 min at 4,400 × *g*) at 16 h post-induction. Periplasmic protein extracts were obtained by osmotic shock. In brief, cells were resuspended in ice cold TES buffer (Tris-HCl 0.2 M, EDTA 0.5 mM, sucrose 0.5 M) and kept for 1 h at 4°C while shaking. Subsequently, TES/4 was added to the cells for 2 h at 4°C while shaking. MgCl_2_ was added to a final concentration of 12 mM and the periplasmic extract was obtained by using the supernatant after two ultracentrifugation steps: first 30 min at 7,860 × *g* and second 15 min at 18,050 × *g*. The extract was $\frac{1}{2}$ diluted in binding buffer (20 mM sodiumphosphate, 0.5 M NaCl and 20 mM imidazole in miliQ) and was loaded onto a Histrap HP column using an Akta Prime plus (GE Healthcare Life Sciences). His-tagged Biv4.40 Nb was eluted using 20 mM sodiumphosphate, 0.5 M NaCl and 0.5 M imidazole. Next, a size exclusion chromatography was performed on a Superdex GF75 10–300 mm column in Gibco LPS-free cell culture grade PBS (ThermoFisher Scientific). Chromatography was performed at 0.5 mL/min with elution of Biv4.40 Nb (30 kDa) after ± 22 min (Supplementary Figure 1A).

### Quality Assessment of the Sn-Binding Capacity of the Bivalent Nanobody

The binding capacity of myc-tagged Biv4.40 to Sn was assessed by immunofluorescent staining. Biv4.40 was added to Sn-expressing CHO cells and non-transfected CHO cells that served as negative controls (30). Cells were seeded onto coverslips and incubated at 37°C for 24 h. Biv4.40 was added to the cells on ice for 60 min at 1 μg/mL. Cells were fixed with 4% paraformaldehyde for 20 min at ambient temperature. After fixation, the secondary mouse-anti-myc antibody (R950-25, ThermoFisher Scientific) was added at 1 μg/mL. After three wash steps with PBS, the chicken anti-mouse Alexa Fluor 488 (ThermoFisher Scientific, A21200) antibody was added to the cells. Nuclei were stained with DAPI (Sigma-Aldrich). Images were obtained using an Axio Observer inverted microscope (Zeiss) equipped with a Compact Light Source HXP 120C and with filter sets 49 and 10 for DAPI and Alexa Fluor 488 fluorophores, respectively. Images were processed using Image J software (Supplementary Figures 1B,C).

### *in vitro* Infections in Bone Marrow-Derived Macrophages

Bone marrow cells from tibia and femur were collected from wildtype and Sn^−/−^ C57BL/6 and BALB/c mice as described previously (31). Red blood cell lysis was performed with ACK buffer for 3 mim. Bone marrow cells were incubated in a petri-dish with 10 mL RPMI-1640 culture medium (Gibco^®^, Life Technologies) enriched with 1% non-essential amino acids, 1% penicillin/streptomycin, 1% sodium pyruvate, 1% L-glutamine, 10% iFCS and 15% L929 cell line supernatant containing macrophage colony stimulating factor (M-CSF) for 6 days at 37°C and 5% CO_2_ (17). On the fourth day of incubation, bone marrow-derived macrophages were either or not stimulated with 50 IU/mL (for assessing dose-dependency) or the standard dose of 5.0 × 10^2^ IU/mL IFN-α (PBL Assay Science, 12100) in a 10 mL petri dish. After incubation, cells were detached with PBS containing 2% 1.0 M HEPES and 1% 0.5 EDTA solution. Bone marrow cells were seeded in 96-well plates at 30,000 cells/well in 100 μL. After 24 h of attachment, cells were infected with *L. infantum* metacyclic promastigotes (multiplicity of infection 5:1) or *ex vivo* amastigotes (multiplicity of infection 20:1) in 100 μL RPMI-1640. In some experiments, extracellular parasites were maximally removed after 24 h of infection, using an established protocol by rinsing the cells 2 × with PBS and incubating the cells with RPMI supplemented with 2% heat-inactivated horse serum, 1% penicillin/streptomycin and 1% L-glutamine (32). After various time points post-infection, infected macrophages were fixed with methanol and stained with Giemsa. For each condition, the intracellular parasite burden was quantified microscopically in at least 50 macrophages for determination of the infection index: $\frac{\#\;amastigotes\;counted}{total\;\#\;macrophages\;counted}$ (33).

### Flow Cytometric Analysis of Sn Expression

Bone marrow cells from tibia and femur were collected and either or not subjected to IFN-α stimulation. Liver cells were collected following a 10-min transcardial perfusion with Krebs-Henseleit solution at a flow rate of 100 mL/h. The gallbladder was removed. Livers were mechanically disrupted in 5 mL DMEM medium (Thermo Fisher) containing liver dissociation enzymes (Miltenyi Biotec) and the gentleMACS™ Dissociator (Miltenyi Biotec). After a 30' enzymatic digestion at 37°C, the cell suspensions were passed through a 100 μm filter. Cells were counted in a KOVA chamber^®^ with trypan blue and resuspended to a concentration of 2.0 × 10^7^ cells/mL. 50 μL (1.0 × 10^6^ cells) of the cell suspension was used for analysis. Cells were kept on ice for 15 minutes and incubated with an Fc-blocking antibody (2.4G2, courtesy Dr. Benoît Stijlemans, VUB, Brussels). Next, cells were stained for 20 minutes with anti-mouse CD169 (Sn)-APC (3D6.112, Biolegend^®^) and the KC panel was supplemented with CD45-APC-Cy7 (30-F11, eBioscienceTM), F4/80-PE-Cy7 (BM8, eBioscience™) and antibodies Tim4-PerCP (RMT4-54, eBioscience™). Viobility 405/520 fixable dye (Miltenyi Biotec) was used for exclusion of dead cells. Flow cytometry was performed on a BD FACSCalibur^®^ apparatus (for bone marrow cells) or MACSQUANT 10^®^ apparatus (for KC) and data were analyzed using the FlowJo^®^ software. The Kupffer cell (KC) gating strategy is presented in Supplementary Figure 2.

### Evaluation of the Impact of Sn and Sialic Acids During *in vitro* Infections

The impact of the Sn-sialic acid interaction was assessed using various complementary approaches. *In vitro* infections were conducted in parallel as described above in bone marrow-derived macrophages from Sn-deficient mice. Alternatively, bone marrow-derived macrophages were pre-incubated with 10 μg/mL SySy94 or Biv4.40 for 1 h prior to infection. To fully exclude any role for LPS despite the precautions taken, cells were treated in one experiment with 25 μg/mL of polymyxin B (81334, Sigma-Aldrich) prior to addition of the pharmacological inhibitors. Blocking of sialic acids on the surface of *Leishmania* parasites was performed by adding 5 μg/mL soluble Sn (5610-SL, Bio-Techne, R&D Systems) to the parasites 1 h prior to infection.

### *in vivo* Infections With VL Strains

Wildtype and Sn-deficient mice were infected in the tail vein with 1.0 × 10^8^ metacyclic promastigotes MHOM/FR/96/LEM3323^PpyRE9^ or MHOM/MA/67/ITMAP^PpyRE9^ in 100 μL RPMI. In some experiments, mice were stimulated subcutaneously (34) with 1,000 IU/g IFN-α 3 days prior to infection. In another experiment, mice were injected intraperitoneally with 4 μg/g Poly(I:C) at 3 days prior to infection, on the day of infection and weekly after infection (35). Poly(I:C) (Sigma-Aldrich, P1530) was freshly dissolved at 800 μg/mL in PBS and was first heated to 50°C for 5 min followed by cooling on ice to maximize annealing.

At different time points post-infection, mice were subjected to bioluminescent imaging in an IVIS^®^ Spectrum *in vivo* Imaging System (PerkinElmer). Briefly, D-luciferin substrate (Promega, Benelux) was injected intraperitoneally (0.15 mg/g BW), followed by anesthesia for 3 min in an induction chamber with 2.5% isoflurane (IsoFlo^®^, Zoetis). Upon induction, mice were imaged in the IVIS^®^ chamber for 10 min. Images were analyzed using LivingImage v4.3.1 within regions of interest (ROI) corresponding with the liver (29).

### Cytokine Response Analysis

Blood samples were collected via the tail vein using heparinized capillaries (75 μL per capillary). Two capillaries were collected per mice. The blood was centrifuged at 20,000 × *g* for 10 min. The supernatant was stored at −80°C until further analysis. A custom panel of cytokines (mouse, IFN-γ, IL-6, IL-10, TNF-α, and KC/GRO) Multispot Assay System kit from MSD^®^ (Mesoscale diagnostics) was used for the multiplex ELISA analysis according to the manufacturer's instructions.

### Statistical Analyses

Mann–Whitney U, Kruskal–Wallis and ANOVA statistical tests were performed in GraphPad Prism 7, considering *p* < 0.05 as statistically significant. Graphs were prepared in GraphPad Prism 7.

## Results

### IFN-α Induces a Higher Susceptibility of Macrophages to Infection With Visceral *Leishmania* Species

**Figure 1:**
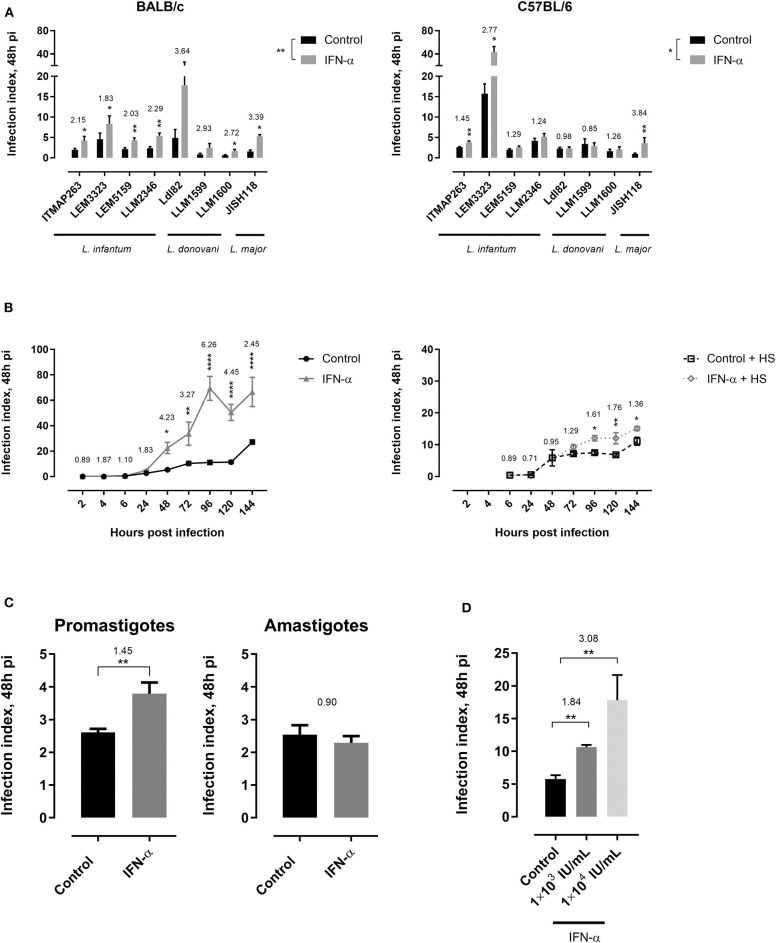
Type I interferon induces a higher susceptibility of macrophages to infection with visceral *Leishmania* species. **(A)** Effect of IFN-α stimulation on the infection index of different *Leishmania* species/strains 48 h post-infection in bone marrow-derived macrophages from BALB/c (left) and C57BL/6 (right) mice. Cells were either left unstimulated (control) or were stimulated with IFN-α 2 days prior to infection with metacyclic promastigotes (5:1 infection ratio). Results in this panel are based on two experiments run in triplicate. **(B)** Effect of IFN-α stimulation on the infection index of *L. infantum* LEM3323 at different time points post-infection in bone marrow-derived macrophages from C57BL/6 mice. Cells were either left unstimulated (control) or were stimulated with IFN-α 2 days prior to infection with metacyclic promastigotes (5:1 infection ratio). Extracellular promastigotes were either washed (right panel) or not (left panel) with PBS 24 h after infection and cells were thereafter incubated with medium containing 2% horse serum (HS). Results in this panel are based on two experiments run in triplicate. **(C)** Infection index in bone marrow-derived cells of C57BL/6 mice either or not stimulated with IFN-α following infection with *L. infantum* ITMAP263 metacyclic promastigote or hamster spleen-derived amastigotes. Results in this panel are based on two experiments run in quadruplicate. **(D)** Effect of different doses of IFN-α stimulation on the infection index of LEM3323 48 h post-infection in bone-marrow derived macrophages from C57BL/6 mice. Results in this panel are based on two experiments run in triplicate. All results are expressed as mean ± standard error of mean (SEM) and the ratio of IFN-α/control is stated above the bars (^*^p < 0.05; ^**^p < 0.01; ^****^p < 0.0001).

We evaluated the infection of different *L. infantum* and *L. donovani* promastigote laboratory strains and clinical isolates in bone marrow-derived macrophages either or not subjected to stimulation with IFN-α. Although the focus of this study was on VL species, the cutaneous *L. major* JISH118 strain was also included. The infection indices were elevated when IFN-α was added to macrophages of BALB/c (*p* = 0.0072) and C57Bl/6 mice (*p* = 0.0480) (Figure 1A). Although some strains seem to benefit more from the IFN-α induced effects than others, recorded infection indices were consistently higher in stimulated macrophages of LEM3323 in both mice species (BALB/c *p* = 0.0180, C57BL/6 *p* = 0.0454), resulting in the selection of this strain for the majority of the subsequent *in vitro* and *in vivo* infection experiments. *In vitro* effects on numbers of intracellular amastigotes were notable within 48 h (Figure 1B, *p* < 0.05). Interestingly, the increase in infection index was mainly due to the cumulative entry of extracellular parasites, rather than an accelerated replication of amastigotes (Figure 1B). Using the *L. infantum* ITMAP263 strain from which both promastigotes and hamster spleen-derived amastigotes were available, the impact of also the life cycle stage could be assessed. In contrast to the promastigote infections (*p* = 0.0047), no effect of IFN-α was observed on infections initiated with ITMAP263 amastigotes under the stated experimental conditions (Figure 1C). The effect of IFN-α was dose-dependent, with the infection index increasing with higher IFN-α concentrations (Figure 1D).

### Upregulation of Sn Expression by IFN-α Enhances *Leishmania* Infection in Macrophages

**Figure 2:**
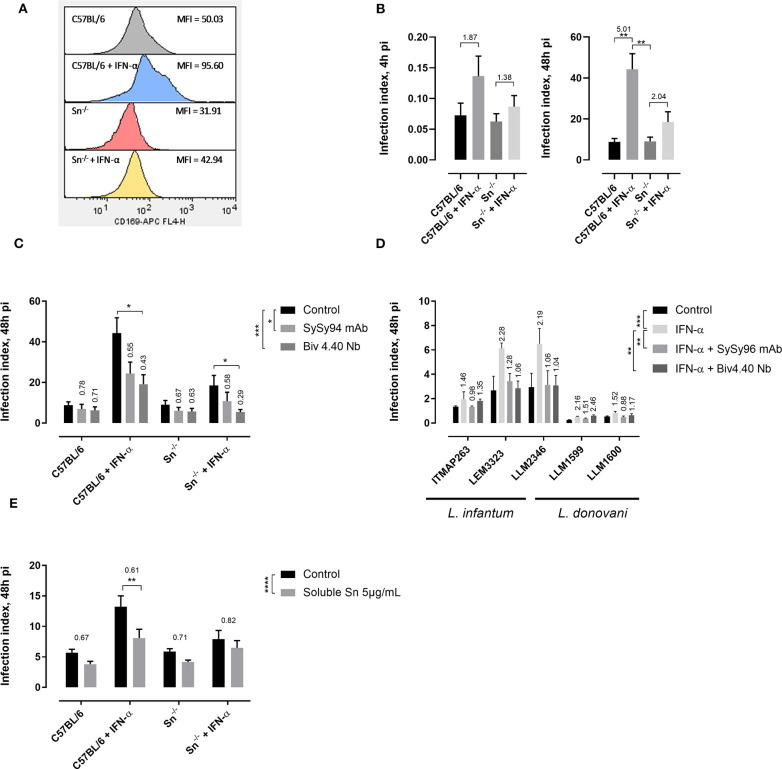
Upregulation of Sn expression by type I interferon enhances *Leishmania* uptake and intracellular multiplication. **(A)** Bone marrow-derived macrophages from C57BL/6 and Sn^−/−^ mice were cultivated for 6 days with or without a 2-day stimulation with 5 × 10^2^ IU/mL IFN-α. Sn surface expression was analyzed by flow cytometry and presented as histograms with the median fluorescence intensity (MFI). These are results from two independent repeats. **(B)** Infection index of LEM3323 promastigote infection (infection ratio 5:1) at 4 and 48 h post-infection in bone marrow-derived macrophages from C57BL/6 and Sn^−/−^ mice either or not pre-stimulated with IFN-α. Results are based on two independent repeats, performed in quadruplicate. The ratio of IFN-α/control is stated above the bars. **(C,D)** Effect of anti-Sn monoclonal antibody SySy94 and anti-Sn Biv4.40 nanobody blocking at a concentration of 10 μg/mL of bone marrow-derived macrophages 1 h prior to infection with **(C)** LEM3323 (infection ratio 5:1) in C57BL/6 mice or **(D)** different *L. infantum* and *L. donovani* strains in BALB/c mice. Results are based on two independent repeats run in triplicate. The ratio of treatment/control is stated above the bars. **(E)** Parasites were incubated with soluble Sn at 5 μg/mL 1 h prior to infection of bone marrow cells. Results were obtained from two independent repeats run in quadruplicate. The ratio of treatment/control is stated above the bars. All results are expressed as mean ± standard error of mean (SEM) (^*^p < 0.05; ^**^p < 0.01; ^***^p < 0.001; ^****^p < 0.0001).

To investigate the impact of IFN-α on Sn expression, a flow cytometric analysis was performed to detect Sn expression (Figure 2A). Incubation of bone marrow-derived macrophages for 2 days with IFN-α resulted in enhanced Sn expression (MFI = 95.6 ± 75.8) compared to the non-stimulated cells (MFI = 50.0 ± 29.8) and IFN-α stimulated Sn^−/−^ cells (MFI = 42.9 ± 21.2).

Since Sn was described to contribute to *Leishmania* entry and multiplication in macrophages (3), the effect of IFN-α stimulation on infection was evaluated in wildtype and Sn^−/−^ macrophages. While marked differences were observed in wildtype macrophages, infection indices did not significantly increase at 4 and 48 h post-infection (*p* > 0.9999) in stimulated bone marrow-derived macrophages originating from Sn^−/−^ mice (Figure 2B). Trends were already notable within 4 h of infection, indicating that IFN-α affects the early infection processes.

The contribution of Sn to the effect of IFN-α was assessed using two different pharmacological inhibitors, a monoclonal anti-Sn antibody (SySy94) and a bivalent nanobody (Biv4.40 Nb) that lacks an Fc antibody domain (Figures 2C,D). Our findings show that pre-treatment of macrophages with Sn-specific antibodies or nanobodies partially abrogates the IFN-α induced effects resulting in a lower infection index 48 h post-infection with the different VL strains (SySy94: *p* = 0.0037; Biv4.40: *p* = 0.0053). Some effects of IFN-α and the pharmacological inhibitors were also noted in Sn^−/−^ mice. Although precautions were taken to purify antibodies and nanobodies in LPS-free conditions, an additional experiment was performed to exclude an impact of LPS by including polymyxin B in the cell system. The same impact of the pharmacological inhibitors on the infection indices was observed in the presence of polymyxin B. To further confirm the role of the Sn-sialic acid interaction, an excess of soluble Sn was added to the parasites prior to infection in order to saturate the surface sialic acids (Figure 2E). A lower infection index (*p* = 0.0013) was obtained in the IFN-α stimulated condition when parasites were pretreated with the soluble Sn compared to the non-treated parasites.

### Sn Plays a Moderate Role During *in vivo* VL Infections

**Figure 3:**
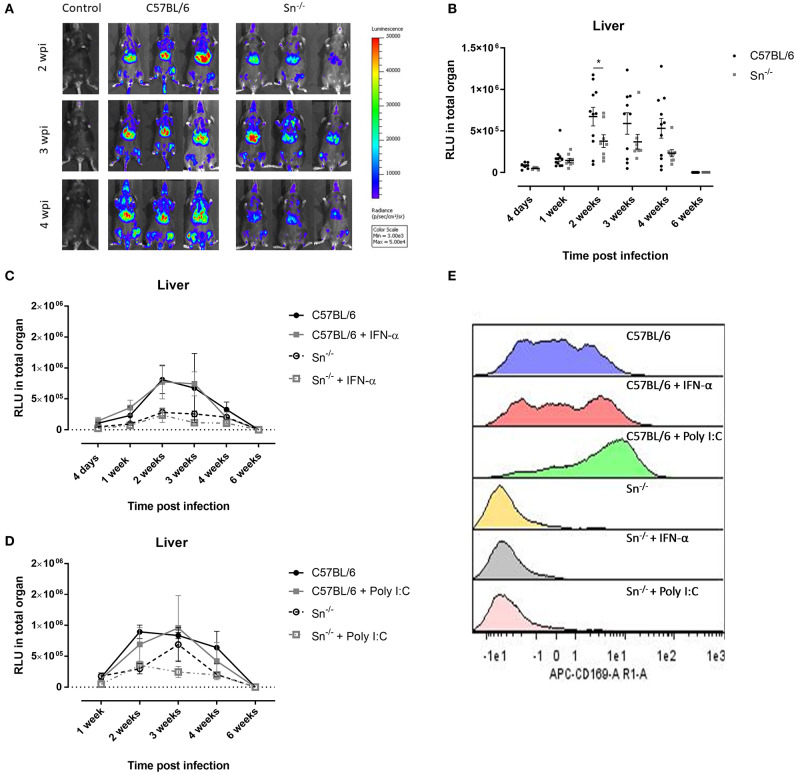
Sialoadhesin plays a moderate role during *in vivo* VL infections. **(A)** Bioluminescent imaging of C57BL/6 and Sn^−/−^ mice infected with *L. infantum* LEM3323^PpyRE9^. Mice were infected with 1.0 × 10^8^ metacyclic promastigotes in the tail vein. These are representative BLI images of infected wildtype and Sn^−/−^ mice of three independent experiments with *n* = 3 mice/group. **(B)** Relative luminescent units (RLU) in a ROI corresponding to the liver as major target organ. Results are based on three independent experiments with *n* = 3 mice/group (^*^p < 0.05). **(C)** Liver burdens in wildtype and Sn^−/−^ mice either or not subjected to a 3–day subcutaneous pre-exposure to 1,000 IU/g IFN-α. Results in this panel are based on *n* = 3 mice/group. **(D)** Liver burdens in wildtype and Sn^−/−^ mice either or not subjected to intraperitoneal inoculation of 4 μg/g Poly(I:C) 3 days prior to infection, on the day of infection and weekly after infection. Results in this panel are based on *n* = 3 mice/group. Results are expressed as mean ± standard error of mean (SEM). **(E)** Sn expression on KCs in control mice and mice treated with IFN-α or Poly(I:C).

Infection of *L. infantum* LEM3323^PpyRE9^ showed higher relative luminescent units in the liver of wildtype as compared to Sn^−/−^ mice at 2 weeks post-infection (*p* = 0.0454) (Figures 3A,B) but was not associated with major changes in the serum cytokine levels (Supplementary Figure 3). Infection with *L. infantum* ITMAP263^PpyRE9^ in wildtype and Sn^−/−^ mice did not show significant differences in liver burdens (Supplementary Figure 4), matching the *in vitro* observations of strain-dependent effects. Additional experiments were performed to mimic an antiviral response by combining the infection with a subcutaneous IFN-α stimulation (Figure 3C) or an exposure to Poly(I:C) (Figure 3D). No significant enhancement of infection was observed in response to these experimental triggers. The impact of these triggers on *in vivo* Sn expression was monitored on KCs (Figure 3E). KCs were found to already express steady-state levels of Sn which are unaffected by the IFN-α stimulation protocol. Poly(I:C) triggers enhanced Sn-expression, but without significantly affecting hepatic parasite burdens (Figure 3D and Supplementary Figure 4C).

## Discussion

The present study provides evidence for an enhanced infection of *Leishmania* in macrophages that are stimulated with type I interferon. Following experimental triggering with IFN-α, bone marrow-derived macrophages become significantly more susceptible to infection by several laboratory and recent clinical VL strains, linked to enhanced promastigote entry. A putative role for type I IFNs that are typically induced during viral infection is in line with recent studies that document an interplay between leishmaniasis and viral infections. For instance, Ethiopian VL patients co-infected with HIV were found to suffer a higher risk of relapse (36). Adaui and colleagues described a significant association between treatment failure and the presence of an endogenous LRV1 in *L. braziliensis* (6). Another study documented that LRV1 confers an advantage to *L. guyanensis* by promoting survival of infected macrophages through a TLR3/miR-155/Akt signaling pathway (37). These combined literature findings suggest that responses induced by HIV-1 or LRV1 can impact on *Leishmania* infection and efficacy of treatment (38).

Blood transcriptomics conducted in *L. infantum* infected individuals revealed activation of the type I interferon pathway (39), which is also observed in infected BALB/c mice (40). Available literature about the role of type I IFN during leishmaniasis seems to ascribe both protective and exacerbating roles. Protective roles of type I IFNs against *L. donovani* relate to interferon regulatory factor-7 (IRF-7) which plays a critical role in regulating amastigote killing (41). Low doses of IFN-β also conferred iNOS-dependent protection of BALB/c mice from progressive cutaneous and fatal visceral disease caused by low and high doses of *L. major* (42). In addition, the *in vivo* protective effects of CpG-oligodesoxynucleotides against *L. major* depend on IFN-α/β-receptor chain 1 (IFNAR1) signaling (43). *In vitro* exposure of mouse peritoneal macrophages to low doses of IFN-α/β and *L. major* promastigotes leads to the expression of iNOS and subsequent killing of intracellular amastigotes. In contrast, high doses and pretreatment exert antagonistic effects on iNOS induction (44). Type I IFN was also reported to correlate with increased susceptibility to *L. infantum* by triggering IL-27 production by macrophages, resulting in inhibition of Th17 responses (45).

Inspired by the effect of IFN-α and the influence of virus presence, the role of the virus-responsive interferon-stimulated gene sialoadhesin (Sn, CD169, Siglec-1) was explored during *Leishmania* infection as Sn is expressed on macrophages in the major VL target organs. In the liver, KC express Sn and are known to function in clearance of microorganisms and senescent cells/debris from the blood (46). Our data in mice confirm that KCs already express Sn in steady-state conditions. In the spleen, Sn^+^ macrophages are a subpopulation of tissue-resident macrophages positioned in the splenic marginal zone that are among the first cell types to encounter invading pathogens (47). These splenic Sn^+^ are often referred to as the marginal-zone metallophilic macrophages (46, 48, 49). In the bone marrow, Sn^+^ macrophages are found in the stroma (19, 50, 51) and have a scavenging function (19, 20). Importantly, all these Sn^+^ macrophage populations become involved during VL infection (52–54). An *in vitro* study further supported a role for sialic acid binding lectins (Siglec-1 and Siglec-5) in *L. donovani* phagocytosis and down-regulation of innate immune signaling responses (3). Next to Sn, other Siglecs were described to be implicated in pathogen-macrophage interactions (3, 55). For *Leishmania* infection, interaction with siglec-5 was shown to deactivate various downstream signaling pathways resulting in a controlled regulation of cytokines in infected macrophages (3).

The present study indicated that the *in vitro* effect of IFN-α is in large part linked to the upregulation of Sn which was confirmed by complementary approaches, i.e., by using Sn-deficient mice, by treatment of macrophages with either an anti-Sn monoclonal antibody or a novel anti-Sn bivalent nanobody (lacking the Fc-domain) and by pretreating parasites with soluble Sn. Both genetic deficiency and pharmacological inhibition largely counteracted the effect of IFN-α. Similarly, Akiyama et al. found that IFN-α stimulation caused enhanced HIV-1 entry and replication in macrophages that could be reduced by pretreatment with an anti-Sn antibody (16). A study on porcine primary alveolar macrophages documented a significant reduction of phagocytotic capacity after Sn-blocking with a monoclonal antibody (56). Some effects of IFN-α and the pharmacological inhibitors were also noted in Sn^−/−^ mice. This supports the implication of additional effects of IFN-α and suggests potential compensatory mechanisms in Sn^−/−^ macrophages (e.g., expression of other Siglecs which may explain promiscuity of the pharmacological inhibitors). For instance, IFN-alpha/beta stimulation of macrophages prior to infection was shown to exert antagonistic effects on iNOS expression (44). IFN-α indeed also slightly enhances susceptibility of Sn^−/−^ macrophages. However, these effects are likely to primarily favor amastigote multiplication, whereas the effects described in this study mainly relate to uptake of the extracellular promastigotes, as illustrated with an established protocol to maximally remove extracellular parasites using horse serum (32).

The role of Sn was further explored *in vivo* by making use of gene-deficient mice (57) and the recently developed bioluminescent *L. infantum* reporter lines LEM3323^PpyRE9^ (29) and ITMAP263^PpyRE9^. Longitudinal follow-up of hepatic parasite burdens in wildtype and deficient mice revealed that Sn only plays a moderate role during *in vivo* VL infections, depending on the *Leishmania* strain used. These results correspond to earlier findings that the surface display of host sialic acids is strain-dependent, resulting in lower virulence when parasites contain fewer sialic acids (3). We also have shown that Sn is already expressed under steady-state conditions on KCs and that neither IFN-α nor poly (I:C) treatment increase liver burdens.

To our knowledge, this is the first study that explored the *in vivo* contribution of Sn to *Leishmania* infection. The role of Sn seems to vary substantially depending on the pathogen involved. Two studies on *Plasmodium* demonstrated that mice depleted of Sn^+^ macrophages developed significantly higher parasitaemia, weight loss and mortality relative to controls (58, 59), indicating that Sn^+^ macrophages are effective in controlling *Plasmodium in vivo*. *Streptococcus pneumonia*, known to replicate inside Sn^+^ splenic macrophages, was not hampered in Sn-deficient mice (60). A study by Martinez-Picado (61) investigated the outcome of HIV-1 in Sn null individuals and found no measurable impact of a truncation in the Sn protein on HIV-1 acquisition or AIDS outcome *in vivo*. This actually contrasts with the *in vitro* functional role of Sn in HIV-1 *trans*-infection. Similarly, our study found a definite functional role *in vitro* but a rather moderate impact *in vivo*. A possible explanation is that Sn primarily affects early infection with promastigotes, but plays a less prominent role during an established infection with amastigotes. The amastigote/promastigote-comparison in our study was limited to the ITMAP263 strain which is not highly modulated by IFN-α but for which hamster spleen-derived amastigotes were available. This could not show elevated uptake and expansion of *L. infantum* ITMAP263 amastigotes in IFN-α stimulated macrophages with elevated surface Sn-expression levels. Amastigotes may indeed employ different strategies for macrophage entry than promastigotes (62), especially because sialic acids on the *Leishmania* surface are acquired by adsorption of serum proteins from the host (23, 24). A study by Chava and colleagues (63) using the Indian *L. donovani* strain MHOM/IN/83/AG83 did demonstrate the presence of sialoglycoconjugates on the amastigote surface, which suggests that strain-dependent differences can be expected.

A recent paper delivered a proof-of-concept that blocking Sn by monoclonal antibodies could be of therapeutic value as exemplified by the halting of Ebola viral uptake and cytoplasmic entry in dendritic cells (64). The fact that Sn null individuals exist also suggests that Sn may serve as a safe therapeutic target (61). Our data on VL indicate that Sn plays a moderate role *in vivo* depending on the parasite strain, although *in vitro* data suggest significant exacerbation in particular conditions with presence of type I interferon. It remains to be seen if the *in vivo* role of the IFN-α/Sn axis could be more prominent under certain conditions, determining whether therapeutic targeting of Sn may deserve further exploration during VL infection and/or in combination with drug treatment.

## Data Availability Statement

All datasets generated for this study are included in the article/Supplementary Material.

## Ethics Statement

The animal study was reviewed and approved by Ethical committee of the University of Antwerp, Belgium.

## Author Contributions

LV, PD, LM, and GC: conceived and designed the experiments. LV, DB, MV, LD, DM, and SH: performed the experiments. LV, DB, and GC: analyzed the data. LV, and GC: wrote the manuscript. PD, LM, and GC: critically revised the manuscript.

## Conflict of Interest

The authors declare that the research was conducted in the absence of any commercial or financial relationships that could be construed as a potential conflict of interest.

## Supplementary Material

Supplementary Figure 1 Purification and binding of Biv4.40 nanobody to Sn-expressing CHO cells. **(A)** Size exclusion chromatography (SEC) profile of Biv4.40 (30 kDa) with an elution peak at 22 min. Abs, absorbance at 280 nm. Biv4.40 binding onto CHO control cells **(B)** and CHO cells expressing mouse sialoadhesin (mSn^+^) **(C)** Cells were stained with Biv4.40 nanobody and anti-myc Alexa Fluor 488 (green) and with DAPI (blue).

Supplementary Figure 2 Gating strategy for KCs. KCs were gated according to their CD45^+^ F4/80^+^ Tim4^+^ expression profile.

Supplementary Figure 3 Cytokine profile over time after infection with LEM3323^PpyRE9^ in wildtype and Sn^−/−^ C57BL/6 mice. Cytokine data of serum samples from C57BL/6 and Sn^−/−^ mice infected in the tail vein with 1.0 × 108 metacyclic promastigotes of *L. infantum* MHOM/FR/96/LEM^3323PpyRE9^. Cytokines **(A)** IL-6, **(B)** IL-10, **(C)** TNF-γ, **(D)** KC/GRO and **(E)** IFN-γ were analyzed with multiplex ELISA. Results in this panel are based two experiments with *n* = 3 mice/group. Results are expressed as mean ± standard error of mean (SEM).

Supplementary Figure 4 Role of Sn during ITMAP263^PpyRE9^ infection. **(A)** Bioluminescent imaging of C57BL/6 and Sn^−/−^ mice infected with *L. infantum* ITMAP^PpYRE9^. Mice were infected with 1.0 × 10^8^ metacyclic promastigotes in the tail vein. **(B)** Relative luminescent units (RLU) in a ROI corresponding to the liver as major target organ. **(C)** Liver burdens in wildtype and Sn^−/−^ mice either or not subjected to intraperitoneal inoculation of 4 μg/g Poly(I:C). Results are expressed as mean ± standard error of mean (SEM).
