# Supplementary figures and images for: Interferon Alpha Favors Macrophage Infection by Visceral Leishmania Species Through Upregulation of Sialoadhesin Expression
Source: Front Immunol. 2020 Jun 9;11:1113. doi: 10.3389/fimmu.2020.01113 (PMC7296180; doi:10.3389/fimmu.2020.01113)

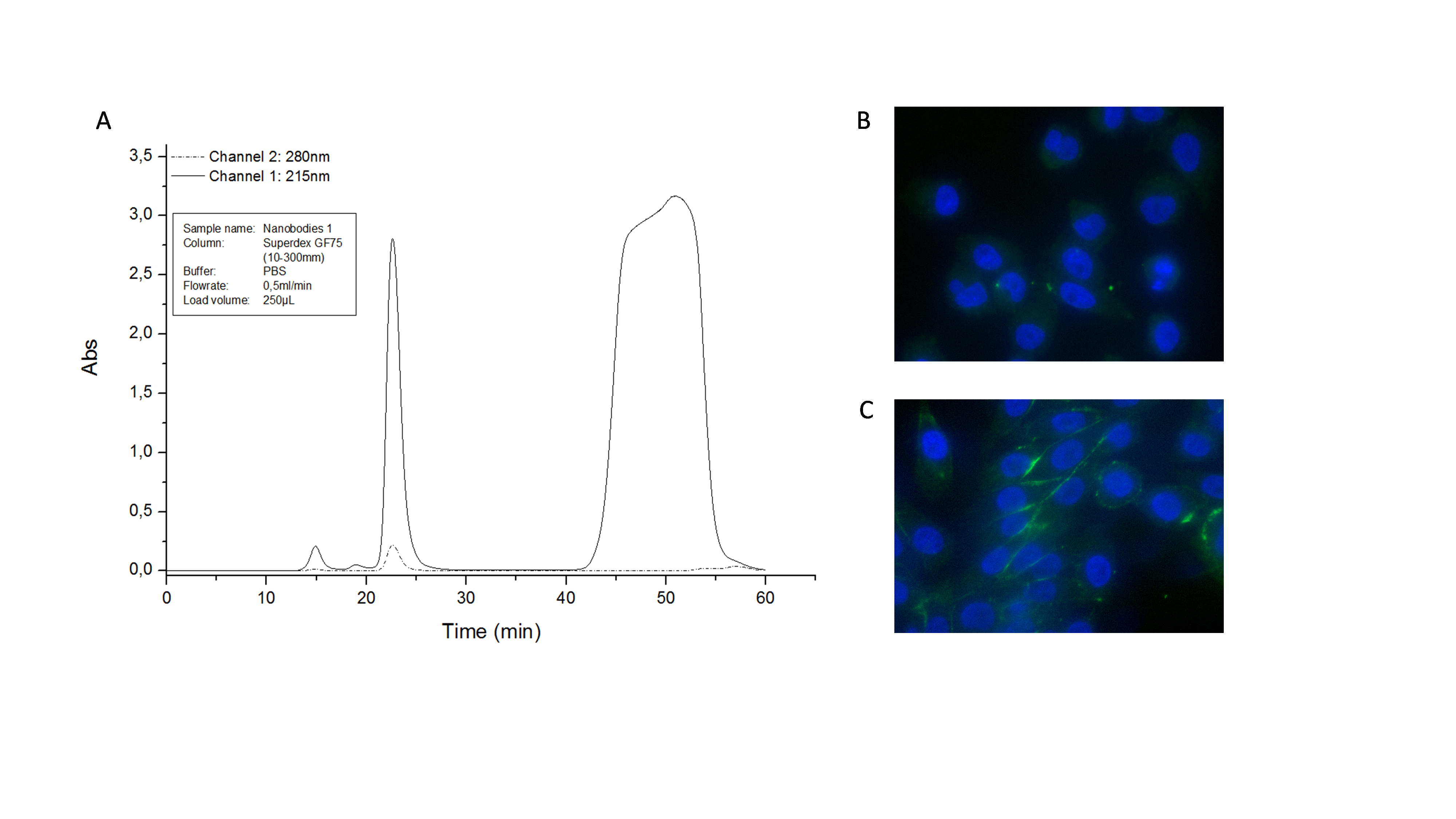

Supplement: Supplementary Figure 1 — Purification and binding of Biv4.40 nanobody to Sn-expressing CHO cells. (A) Size exclusion chromatography (SEC) profile of Biv4.40 (30 kDa) with an elution peak at 22 min. Abs, absorbance at 280 nm. Biv4.40 binding onto CHO control cells (B) and CHO cells expressing mouse sialoadhesin (mSn+) (C) Cells were stained with Biv4.40 nanobody and anti-myc Alexa Fluor 488 (green) and with DAPI (blue). [file Image_1.TIFF]

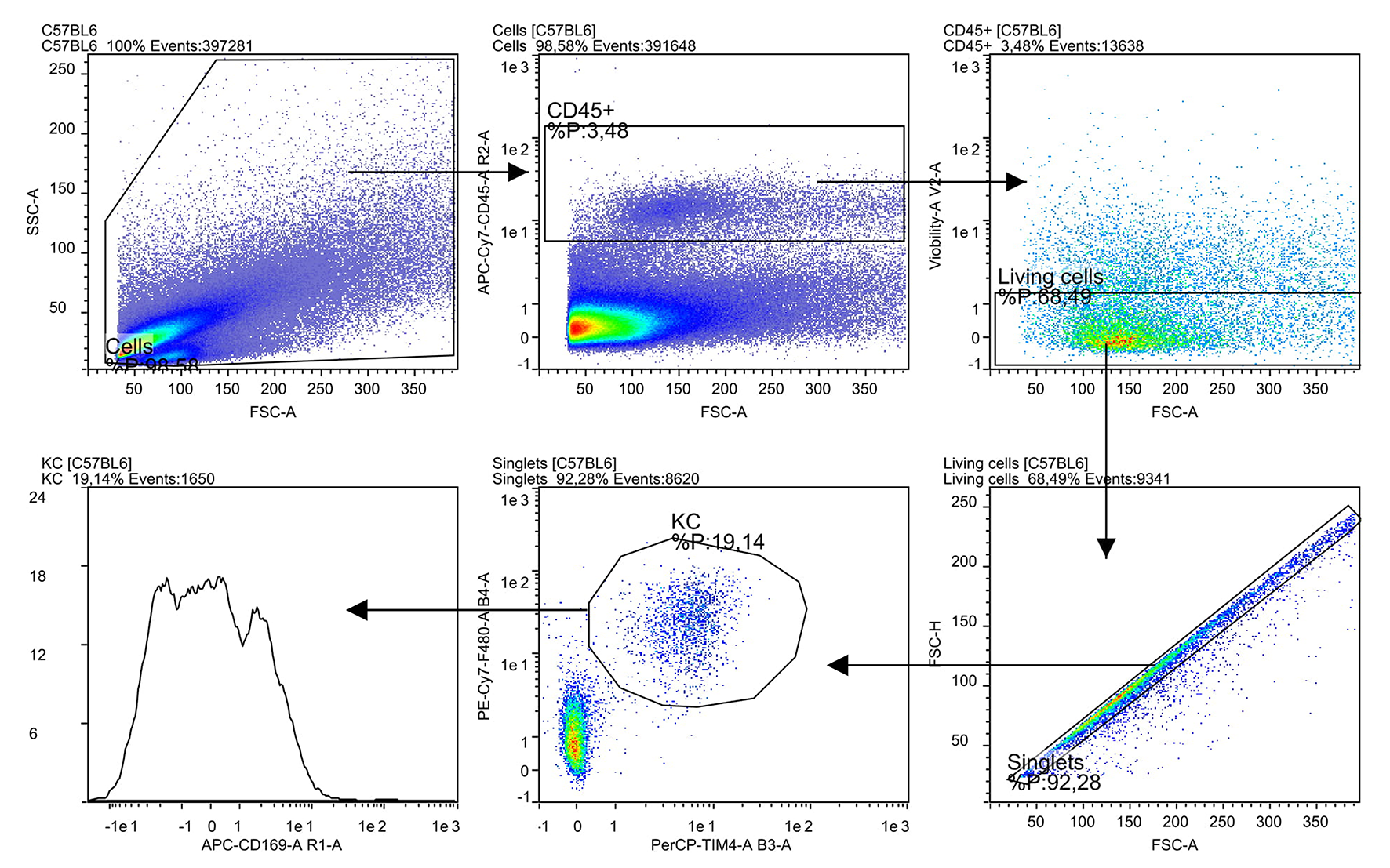

Supplement: Supplementary Figure 2 — Gating strategy for KCs. KCs were gated according to their CD45+ F4/80+ Tim4+ expression profile. [file Image_2.TIFF]

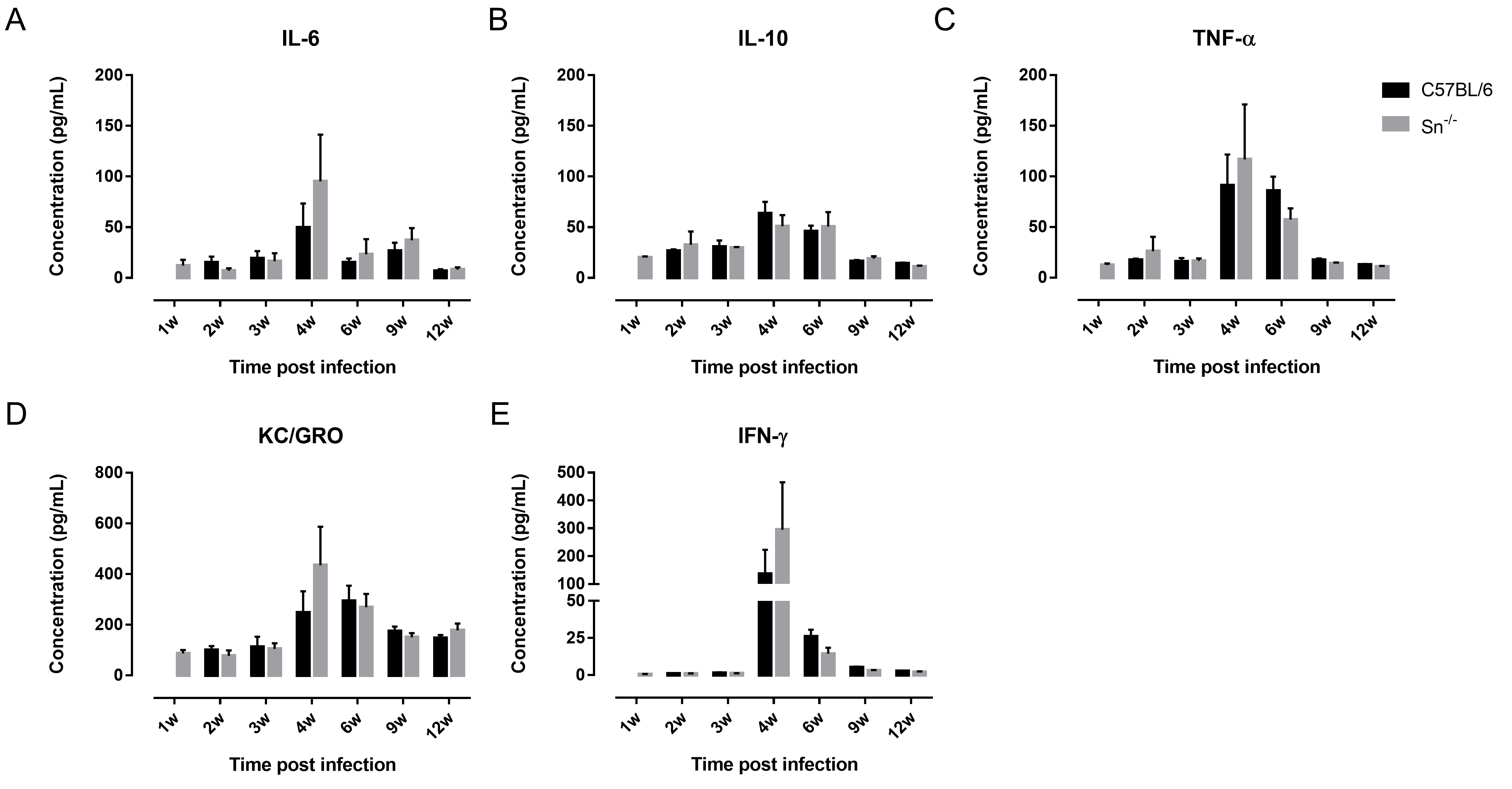

Supplement: Supplementary Figure 3 — Cytokine profile over time after infection with LEM3323PpyRE9 in wildtype and Sn−/− C57BL/6 mice. Cytokine data of serum samples from C57BL/6 and Sn−/− mice infected in the tail vein with 1.0 × 10^8^ metacyclic promastigotes of L. infantum MHOM/FR/96/LEM3323PpyRE9. Cytokines (A) IL-6, (B) IL-10, (C) TNF-γ, (D) KC/GRO and (E) IFN-γ were analyzed with multiplex ELISA. Results in this panel are based two experiments with n = 3 mice/group. Results are expressed as mean ± standard error of mean (SEM). [file Image_3.TIFF]

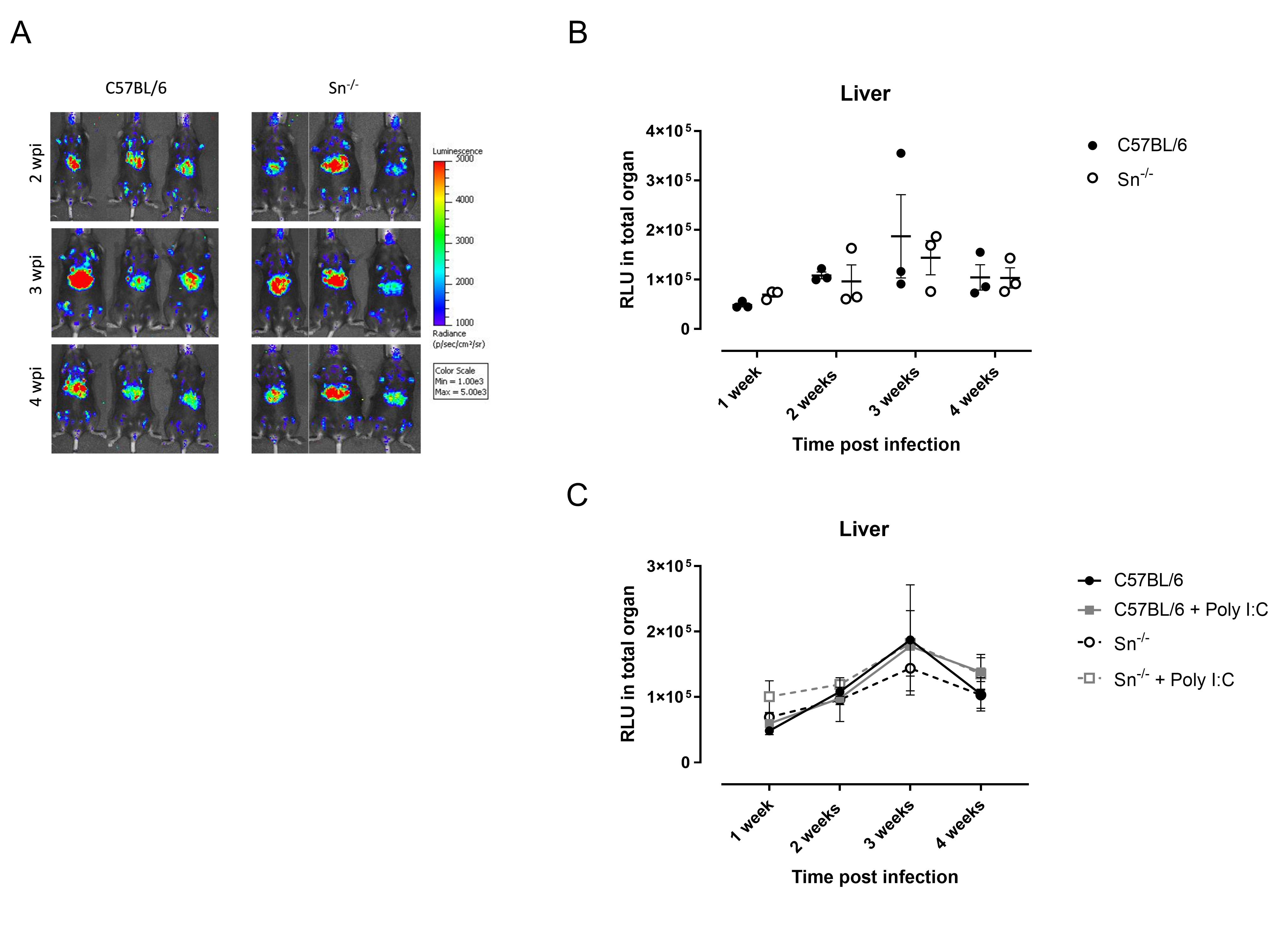

Supplement: Supplementary Figure 4 — Role of Sn during ITMAP263PpyRE9 infection. (A) Bioluminescent imaging of C57BL/6 and Sn−/− mice infected with L. infantum ITMAPPpYRE9. Mice were infected with 1.0 × 10^8^ metacyclic promastigotes in the tail vein. (B) Relative luminescent units (RLU) in a ROI corresponding to the liver as major target organ. (C) Liver burdens in wildtype and Sn−/− mice either or not subjected to intraperitoneal inoculation of 4 μg/g Poly(I:C). Results are expressed as mean ± standard error of mean (SEM). [file Image_4.TIFF]
